# Complete mitochondrial genomes of the charr *Salvelinus alpinus erythrinus* (Salmoniformes: Salmonidae) from Arctic Canada

**DOI:** 10.1080/23802359.2021.2023334

**Published:** 2022-01-24

**Authors:** Alla G. Oleinik, Andrey D. Kukhlevsky, Lubov A. Skurikhina

**Affiliations:** A.V. Zhirmunsky National Scientific Center of Marine Biology, Far Eastern Branch, Russian Academy of Sciences, Vladivostok, Russia

**Keywords:** mtDNA, charr genus *Salvelinus*, Arctic charr, Taranetz charr, *Salvelinus taranetzi*

## Abstract

The complete mitochondrial genomes were sequenced in two individuals of charr *Salvelinus alpinus erythrinus* from Arctic Canada. The genome sequences were 16,652 bp in length; the genome organization and GC content (45.6%) are consistent with those of charr mitochondrial genomes published previously. The difference between the two genomes studied is low, 0.02%. Our results indicate the phylogenetic closeness of *S. alpinus erythrinus* and *Salvelinus* sp. 4 from Lake Nachikinskoe, Kamchatka and their origin from a common ancestor.

The Arctic charr *Salvelinus alpinus* Linnaeus, 1758 (or the *S. alpinus* complex) is widely distributed in the northern Holarctic (Reist et al. 2013). There are several subspecies that were commonly proposed (*S. alpinus alpinus*, *S. alpinus erythrinus*, and *S. alpinus oquassa*) based on their zoogeographic patterns and morphological characteristics (Taylor 2016 and references therein). We recently sequenced the complete mitochondrial genomes (mitogenomes) of *S. alpinus alpinus* (Oleinik et al. 2020) and *S. taranetzi* (Oleinik et al. 2019b). In this study, we examined the mitogenomes of *S. alpinus erythrinus* (synonyms *S. alpinus stagnalis*, *S. alpinus taranetzi*) from Arctic Canada and compared them with genomes of charrs from East Asia to North America to assess the phylogeographic patterns of charr.

We sequenced and described two mitogenomes of *S. alpinus erythrinus* from Jayko River, Victoria Island, Nunavut, Canada (69°42.56′ N, 103°16.82′ W). The fish specimens are stored in the collection of the Genetics Laboratory, NSCMB FEB RAS, Vladivostok, Russia (www.imb.dvo.ru) under accession numbers JAY11.J2 and JAY11.J3. Total genomic DNA was isolated from fin fragments using standard phenol–chloroform extraction and ethanol precipitation methods (Sambrook and Russell 2001). In order to amplify the mitogenomes, 23 pairs of primers (Table S1) were designed with the MitoPrimer_V1 (Yang et al. 2011) based on mitogenomes of charr species public in GenBank. Amplified DNA was purified using ExoSap-IT (Thermo Fisher Scientific, Waltham, MA) and directly sequenced using a BigDye Terminator Cycle Sequencing Ready Reaction Kit 3.1 (Applied Biosystems, Foster City, CA) by GA3500 automated sequencer (Applied Biosystems, Foster City, CA). The sequenced fragments were assembled into genomes and annotated by comparing with published mitogenome sequences of charr using Geneious R11 (http://www.geneious.com/). The mitogenomes of *S. alpinus* from northeastern Québec, Canada (GenBank accession number NC_000861; *S. alpinus oquassa* sensu Oleinik et al. 2019a), and *S. alpinus alpinus* (MN957795 and MN957796) from the Scandinavian Peninsula were included for comparison. Nucleotide content, codon position, the average number of nucleotide substitutions per site between groups (the total sequence divergence, *Dxy*), were calculated using MEGA X (Kumar et al. 2018).

The mitogenome of *S. alpinus erythrinus* was 16,652 bp in length. The genome organization was identical to that of typical salmon mitogenomes, including two ribosomal RNA (rRNA) genes, 13 protein-coding genes (PCGs), 22 transfer RNA (tRNA) genes, a light-strand replication origin (OL), and a control region (CR). The overall base composition was 28.0% A, 26.4% T, 17.0% G, and 28.6% C, and the GC content of the mitogenomes was 45.6%. This is consistent with previous results for charr mitogenomes (Balakirev et al. 2016; Oleinik et al. 2019b). We detected three single-nucleotide substitutions and one sequence length difference between *S. alpinus erythrinus* (MW664921) and *S. alpinus erythrinus* (MW664922); the total sequence divergence was 0.000180 ± 0.000097. There were 193 single-nucleotide substitutions (in the CR, tRNA, 12S rRNA, 16S rRNA, and PCGs) between mitogenome sequences of *S. alpinus erythrinus* and *S. alpinus alpinus*, and a length difference due to an insertion (GA) in the non-coding region (*OL*). Of these, 145 substitutions were found in overall PCGs, and 31 substitutions in CR. A similar distribution of 190 single-nucleotide substitutions was observed when comparing mitogenomes of *S. alpinus erythrinus* and *S. alpinus oquassa* (NC_000861): 147 substitutions in PCGs and 29 substitutions in CR. Protein coding genes had a different degree of variability, but variability of the NADH dehydrogenase subunit genes was highest for the mitogenomes compared (68.9% and 66.7% of all variable sites in PCGs for two pairs of comparison).

The comparison of mitogenomes now obtained with 27 mitogenomes of related groups available in GenBank including genera *Salvelinus*, *Parahucho*, and *Salmo* indicates a close affinity of *S. alpinus erythrinus* to a congeneric species, *S. taranetzi* (and closely related taxa) (Figure 1). The difference (*Dxy*) between them was 0.00278 ± 0.00033, which is in close agreement with the values of intraspecific divergence previously reported for *S. taranetzi* (Oleinik et al. 2015, 2019a). The total sequence divergence between *S. alpinus erythrinus* and *Salvelinus* sp. 4 from Lake Nachikinskoe, Kamchatka (MW181766 and MW181767) is lowest, 0.0011 ± 0.0002. The *S. alpinus erythrinus* mitogenomes studied here showed similar sequence divergence (0.0110 ± 0.0007 on average) from the GenBank mitogenomes of *S. alpinus alpinus* and *S. alpinus oquassa* (NC_000861). These values correspond to the level of intraspecific variability in the genus (Oleinik et al. 2015). In general, this is consistent with the previous results obtained using individual genes: *Cytb*, *COI*, and CR (Oleinik et al. 2017, 2019a; Esin et al. 2017). This degree of divergence supports recent divergence of *S. alpinus erythrinus* and *Salvelinus* sp. 4 and/or their origin from a common ancestor. Undoubtedly, members of Arctic charr phylogenetic group from North America (*S. alpinus erythrinus* (syns. *S. alpinus stagnalis*, *S. alpinus taranetzi*)) and North East Asia belong to the same species that was originally described as *S. taranetzi* Kaganovsky, 1955 from Lake Achchen (Chukotka).

**Figure 1.**
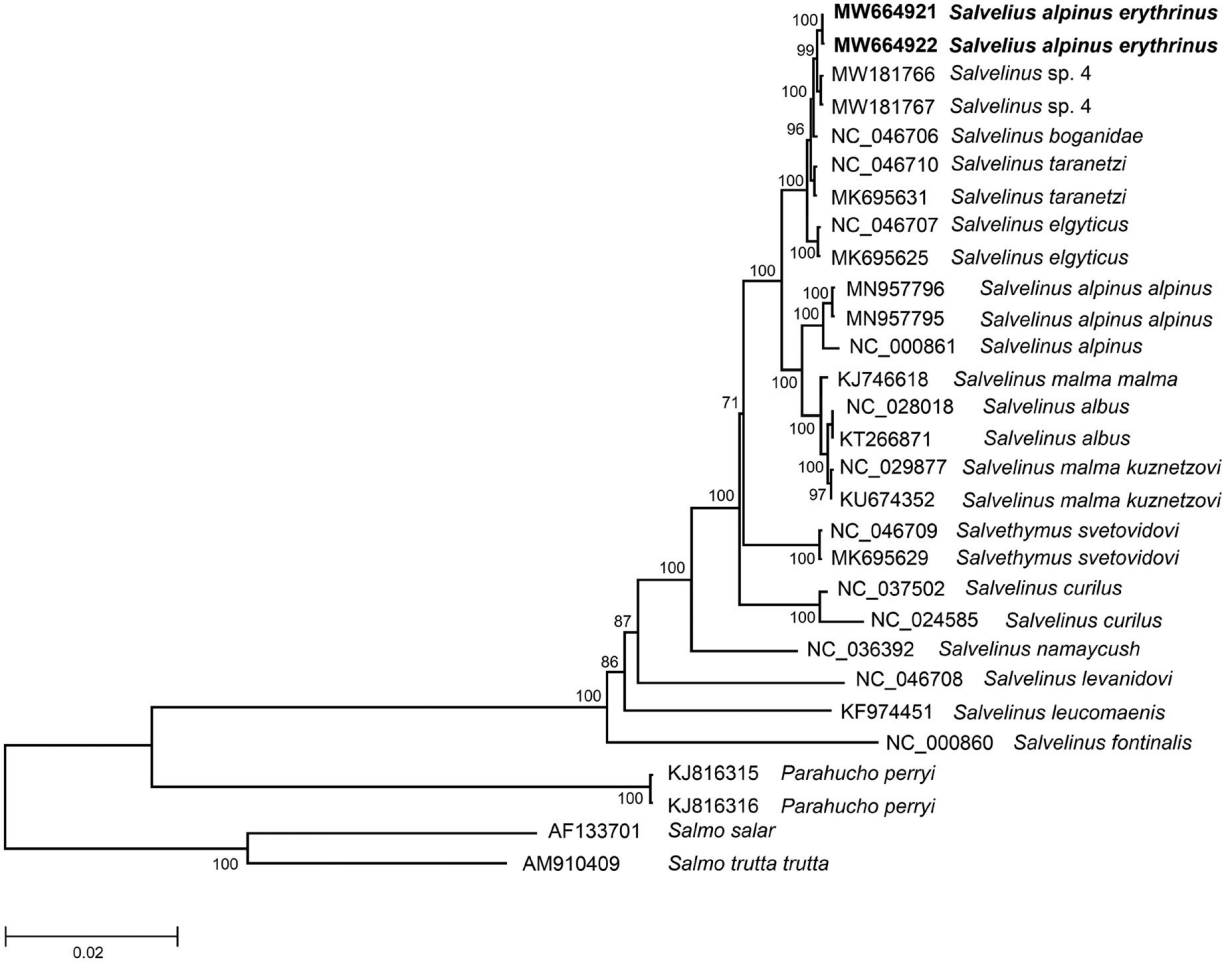
Maximum-likelihood (ML) tree constructed from comparison of complete mitogenome sequences of *Salvelinus alpinus erythrinus* and other GenBank representatives of the family Salmonidae. The tree is based on the GTR plus gamma plus invariant sites (GTR + G+I) model of nucleotide substitution. GenBank accession numbers for all sequences are listed in the figure. Numbers at the nodes indicate bootstrap probabilities from 1000 replications. The mitogenome sequences of *Salvelinus alpinus erythrinus* in this study are marked in bold. Phylogenetic analysis was conducted in MEGA X (Kumar et al. 2018).

## Supplementary Material

Supplemental Material

## Data Availability

The data that support the findings of this study are openly available in the National Center for Biotechnology Information database (NCBI/GenBank) at https://https.ncbi.nlm.nih.gov/, reference numbers MW664921 and MW664922.
