# Supplementary material for: Complete mitochondrial genomes of the charr Salvelinus alpinus erythrinus (Salmoniformes: Salmonidae) from Arctic Canada
Source: Mitochondrial DNA B Resour. 2022 Jan 24;7(1):231–3. doi: 10.1080/23802359.2021.2023334 (PMC8788382; doi:10.1080/23802359.2021.2023334)
Supplement: Supplemental Material [file TMDN_A_2023334_SM5304.docx]

**SUPPORTING INFORMATION**

**Complete mitochondrial genomes of endemic charr of the genus *Salvelinus* from Lake Nachikinskoe (Kamchatka, Russia)**

Alla G. Oleinik, Andrey D. Kukhlevsky, Lubov A. Skurikhina

Correspondence

Alla G. Oleinik, A.V. Zhirmunsky National Scientific Center of Marine Biology, Far Eastern Branch, Russian Academy of Sciences, Vladivostok, Russia.

E-mail: [alla_oleinik@mail.ru](mailto:alla_oleinik@mail.ru)

**Table S1 Primers used in the present study for the charr of the genus *Salvelinus***

| Primer [1]  Forward: CAAGTCTCCGCATTCCTGTGAG  Reverse: GAGGTGCTTTCCTATTTTAGCTACA | Start: 119  Start: 1045 | Length: 22 Length: 25 | Tm: 62  Tm: 59 | GC%: 54  GC%: 40 |
| --- | --- | --- | --- | --- |
| Primer [2]  Forward: TATACCACCGTCGTCAGCTTACC  Reverse: AGGTTGTGTCCTTGTTCAAAGGAG | Start: 678  Start: 1604 | Length: 23 Length: 24 | Tm: 61  Tm: 62 | GC%: 52  GC%: 46 |
| Primer [3]  Forward: GTTATAGCTGGTTGCTTAGGAAATG  Reverse: TCAGAAATTCTGGTGCTTAGAGC | Start: 1463  Start: 2415 | Length: 25 Length: 23 | Tm: 59  Tm: 59 | GC%: 40  GC%: 44 |
| Primer [4]  Forward: CTTTAGACACCAGGCAGATCACG  Reverse: GTAAGTGCAAGTGTGAGGGCAAG | Start: 2239  Start: 3110 | Length: 23 Length: 23 | Tm: 62  Tm: 62 | GC%: 52  GC%: 52 |
| Primer [5]  Forward: CAGAGGTTCAAACCCTCTCCTTAG  Reverse: TTAAGCATTCAGGCACAATTCC | Start: 2816  Start: 3844 | Length: 24 Length: 23 | Tm: 61  Tm: 60 | GC%: 50  GC%: 41 |
| Primer [6]  Forward: TAGCACTTGTCCTATGACACCTC  Reverse: CAGTGCAAGGAATGCTGAAG | Start: 3752  Start: 4687 | Length: 23 Length: 20 | Tm: 56  Tm: 57 | GC%: 48  GC%: 50 |
| Primer [7]  Forward: ATACTGGCCCTTGCACTTAAAC  Reverse: TTTGTAGGATCGAGGCCTTCC | Start: 4325  Start: 5251 | Length: 22 Length: 21 | Tm: 58  Tm: 61 | GC%: 46  GC%: 52 |
| Primer [8]  Forward: CTTCAAAGCTCTAAGCGGGAGTG  Reverse: TGTTTAGATTTCGGTCCGTGAGTAAC | Start: 5112  Start: 6120 | Length: 23 Length: 26 | Tm: 63  Tm: 63 | GC%: 52  GC%: 42 |
| Primer [9]  Forward: AACCCCGCTTTTTGTTTGAG  Reverse: CGATTGACGCTACTTCTCGTTTG | Start: 6011  Start: 6927 | Length: 20 Length: 23 | Tm: 60  Tm: 62 | GC%: 45  GC%: 48 |
| Primer [10]  Forward: CCGGACGCCTACACACTATG  Reverse: TAAAGGCTGTTTGGTTTAATCG | Start: 6801  Start: 7738 | Length: 20  Length: 22 | Tm: 59  Tm: 57 | GC%: 60  GC%: 36 |
| Primer [11]  Forward: AATGGGCCACCAATGATATTG  Reverse: TGCAAGGCCTATATTTAGGGAGAG | Start: 7482  Start: 8427 | Length: 21 Length: 24 | Tm: 60  Tm: 62 | GC%: 43  GC%: 46 |
| Primer [12]  Forward: GGTTCATCAACCGATTTACTCA  Reverse: AATACTTCGGAGGTAATAAATAAGA | Start: 8255  Start: 9067 | Length: 22 Length: 25 | Tm: 57  Tm: 54 | GC%: 41  GC%: 28 |
| Primer [13]  Forward: GCAGTCTGATTCCACTTCCAC  Reverse: AATAGGATGGCGATTAGGAAG | Start: 8886  Start: 9835 | Length: 21 Length: 21 | Tm: 57  Tm: 56 | GC%: 52  GC%: 41 |
| Primer [14]  Forward: AGCCACTATTTCTTTCTGATTACC  Reverse: TCGGTTGCTAGATAAAGGTTAGAG | Start: 9701  Start: 10535 | Length: 24 Length: 24 | Tm: 57  Tm: 58 | GC%: 38  GC%: 42 |
| Primer [15]  Forward: TAGCTCCCATACTTCTGTTAGCA  Reverse: AGTGATTTCAGGTCCGTTTGACG | Start: 10239  Start: 11213 | Length: 23 Length: 23 | Tm: 57  Tm: 63 | GC%: 43  GC%: 48 |
| Primer [16]  Forward: CACGTAGAAGCCCCAATCGC  Reverse: AGGGTGGTGATAAGGGGGTAAGT | Start: 11017  Start: 12029 | Length: 20 Length: 23 | Tm: 63  Tm: 62 | GC%: 60  GC%: 52 |
| Primer [17]  Forward: CAAAAACTCTTGGTGCAAATCC  Reverse: ACGATTTTTTTGATGTCGTTTTG | Start: 11917  Start: 12872 | Length: 22 Length: 22 | Tm: 59  Tm: 59 | GC%: 41  GC%: 30 |
| Primer [18]  Forward: GTATCTGCCCTACTTCACTCTAGC  Reverse: CTATCTGGCTGGCAATAGTTTG | Start: 12694  Start: 13627 | Length: 24 Length: 22 | Tm: 57  Tm: 57 | GC%: 50  GC%: 46 |
| Primer [19]  Forward: ACTTCCTGCCCTCCAAAACCCC  Reverse: ACCCTGCCCCATACTTTGCTGC | Start: 13379  Start: 14242 | Length: 22 Length: 22 | Tm: 67  Tm: 65 | GC%: 59  GC%: 59 |
| Primer [20]  Forward: ACGATCCCCCCAAGACTCAG  Reverse: AAGTACTGTGGCGGCTGCAA | Start: 14052  Start: 14963 | Length: 20 Length: 20 | Tm: 61  Tm: 62 | GC%: 60  GC%: 55 |
| Primer [21]  Forward: ACGCTAACGGAGCATCTTTC  Reverse: TAAAATCTCCTCTCTCTGAGCAC | Start: 14626  Start: 15609 | Length: 20 Length: 23 | Tm: 57  Tm: 55 | GC%: 50  GC%: 43 |
| Primer [22]  Forward: TGCCTCTGTGATTTACTTCACCATC  Reverse: TGGTTTAGGGGTTTAACAGGAAC | Start: 15437  Start: 16472 | Length: 25 Length: 23 | Tm: 62  Tm: 60 | GC%: 44  GC%: 43 |
| Primer [23]  Forward: GTAAAGCATCTGGTTAATGGTG  Reverse: ATCTAATCCCAGTTTGTGTCGTAG | Start: 16130  Start: 499 | Length: 22 Length: 24 | Tm: 55  Tm: 58 | GC%: 41  GC%: 42 |

*Note.* The primers are designed with the program MitoPrimerV1 (Yang et al. 2011). Start of reading (Start) is indicated for forward and reverse primers according to complete mitochondrial genome of *Salvelinus taranezi* isolat TRAC95.070 (NC_046710).

**References**

Yang C-H, Chang H-W, Ho C-H, Chou Y-C, Chuang L-Y. 2011. Conserved PCR primer set designing for closely-related species to complete mitochondrial genome sequencing using a sliding window-based PSO algorithm. PLoS ONE 6(3): e17729.
